# Magnetic Resonance Imaging of Human-Derived Amniotic Membrane Stem Cells Using PEGylated Superparamagnetic Iron Oxide Nanoparticles

**DOI:** 10.22074/cellj.2016.4560

**Published:** 2016-08-24

**Authors:** Maryam Naseroleslami, Kazem Parivar, Samideh Khoei, Nahid Aboutaleb

**Affiliations:** 1 Department of Biology, Science and Research Branch, Islamic Azad University, Tehran, Iran; 2 Department of Medical Physics, School of Medicine, Iran University of Medical Sciences, Tehran, Iran; 3 Physiology Research Center, Iran University of Medical Sciences, Tehran, Iran; 4 Department of Physiology, Iran University of Medical Sciences, Tehran, Iran

**Keywords:** Imaging, Nanoparticles, Stem Cells

## Abstract

**Objective:**

The label and detection of cells injected into target tissues is an area of focus for researchers. Iron oxide nanoparticles can be used to label cells as they have special characteristics. The purpose of this study is to examine the effects of iron oxide nanoparticles on human-derived amniotic membrane stem cell (hAMCs) survival and to investigate the magnetic properties of these nanoparticles with increased contrast in magnetic resonance imaging (MRI).

**Materials and Methods:**

In this experimental study, we initially isolated mesenchymal stem cells from amniotic membranes and analyzed them by flow cytometry. In addition, we synthesized superparamagnetic iron oxide nanoparticles (SPIONs) and characterized them by various methods. The SPIONs were incubated with hAMCs at concentrations of 25-800 μg/mL. The cytotoxicity of nanoparticles on hAMCs was measured by the MTT assay. Next, we evaluated the effectiveness of the magnetic nanoparticles as MRI contrast agents. Solutions of SPION were prepared in water at different iron concentrations for relaxivity measurements by a 1.5 Tesla clinical MRI instrument.

**Results:**

The isolated cells showed an adherent spindle shaped morphology. Polyethylene glycol (PEG)-coated SPIONs exhibited a spherical morphology. The average particle size was 20 nm and magnetic saturation was 60 emu/g. Data analysis showed no significant reduction in the percentage of viable cells (97.86 ± 0.41%) after 72 hours at the 125 μg/ml concentration compared with the control. The relaxometry results of this SPION showed a transverse relaxivity of 6.966 (μg/ml.s)^-1^

**Conclusion:**

SPIONs coated with PEG used in this study at suitable concentrations had excellent labeling efficiency and biocompatibility for hAMCs.

## Introduction

Cell therapy has been suggested as the best way to treat most diseases. Thus far, a wide range of stem cells has been employed to enhance tissue damage repair in animal as well as human tissues. Amniotic membrane stem cells are particularly interesting because of their ease of preparation, lack of stimulation of the immune system, high potency for differentiation, and secretion of growth and anti-inflammatory factors (1). 

In addition to the cell type, tracking the cell after injection into the target tissue plays an important role in cell therapy (2). Although numerous methods are used to detect these cells, iron oxide nanoparticles are most common because they can enter cells without intervention and are nontoxic at certain concentrations (3). Because of their high sensitivity and appropriate size, iron oxide nanoparticles are used as contrast agents in magnetic resonance imaging (MRI) (2). 

MRI is recognized as a commonly used, strong diagnostic technique in medical fields. It is noninvasive, easy to use, and has a high penetration to obtain detailed internal cross-sectional images of living organisms (4). Biological tissues have aqueous environments. The signal intensity of MRI depends on the local values of longitudinal or transverse relaxation rate of water protons (5). Application of contrast agents plays a significant role by enhancing the contrast between tissue types by increasing the image quality, which therefore increases the sensitivity of the MRI method (6,7). 

MRI is recognized as a commonly used, strong diagnostic technique in medical fields. It is noninvasive, easy to use, and has a high penetration to obtain detailed internal cross-sectional images of living organisms (4). Biological tissues have aqueous environments. The signal intensity of MRI depends on the local values of longitudinal or transverse relaxation rate of water protons (5). Application of contrast agents plays a significant role by enhancing the contrast between tissue types by increasing the image quality, which therefore increases the sensitivity of the MRI method (6, 7). Two different classes are used as contrast agents in MRI-T_1_ agents that decrease proton longitudinal relaxation time providing positive contrast (gadolinium complexes or manganese ions) and T_2_ agents that truncate proton transverse relaxation time providing negative contrast (iron oxide nanoparticles). Superparamagnetic iron oxide nanoparticles (SPIONs), are relatively safe T_2_ contrast agents for MRI with excellent sensitivity (8,9). 

Because of their unique characteristics nanoparticles are used for cell detection. However some studies suggest that these nanoparticles decrease cell proliferation, induce apoptosis, inflammation, DNA damage, and oxidative stress in cells (10,12). The toxicity of SPION is strongly associated with the dose and coating of these nanoparticles. The choice of more biocompatible materials for coating the magnetic nanoparticles can resolve the problem of cytotoxicity (13,14). In order to decrease the cytotoxic effects of nanoparticles, it is possible to coat their surface with various polymers and biomolecules (15). Polyethylene glycol (PEG) is one of the preferred materials for coating magnetic nanoparticles due to its high bio-stability and very low toxicity. PEG has extensive applications in medicine (16,17). 

In this study, we used an appropriate dose of a PEG-coated SPION, a biocompatible coat, to label amniotic membrane stem cells. We investigated the role of SPION in terms of MRI quality. The results of this study might help to use MRI as a monitor of labeled hAMCs injected into damaged tissues for possible future use in cell therapy. 

## Materials and Methods

### Synthesis and characterization of superparamagnetic iron oxide nanoparticles

In this experimental study, we synthesized SPION samples by co-precipitation. SPIONs were synthesized by deposition of alkaline salts of iron ions through a single-step process. 

First, the hydrated iron chlorides, FeCl_2_.4HO_2_ and FeCl_3_.6HO_2_ were dissolved in distilled water at a 2:1 ratio, using a stirrer and deoxidized by nitrogen at room temperature. Then, ammonium hydroxide was dropped into this solution, while under stirring conditions and the pH increased to 11. The addition of ammonium hydroxide resulted in a brown sediment which was removed by a magnet after 30 minutes of stirring. Sediment has been rinsed and separated several times by distilled water and ethanol. It was dried at room temperature after two acetone washes. In order to determine the particle size and morphology, we used high resolution transmission electron microscopy (HR-TEM). The particles were coated with 3-amino propyl tri ethoxyl silane (APTES) so that carboxyl polyethylene could bind to the surface amine. We investigated the magnetic property by using vibrating sample magnetometer (VSM).

## Informed consent

We obtained written permission from pregnant women who were hospitalized in Milad Hospital, Tehran, Iran. Participants were informed about the research study and were assured that there would be no harm to their delivery process. 

## Cell culture

We confirmed the presence of these stem cells by performing differential tests. Cells were extracted from the newborns’ amniotic sacs as published in our previous study (18). The cells were maintained in complete Dulbecco’s Modified Eagle’s Medium (DMEM) supplemented with 10% fetal bovine serum (FBS), NaHCO_3_ (3.7 g/l), penicillin (100 U/ ml), and streptomycin (100 mg/ml, Sigma, USA). Cells were then grown in a humidified atmosphere of 5% CO_2_ and 95% air in an incubator at 37˚C. When the cells reached 70-80% confluency, they were used for labeling with SPIONs. When the cells reached 70-80% confluency, they were used for labeling with SPIONs. 

## Cell characterization by flow cytometry 

In order to ensure that the isolated cells were mesenchymal cells, we performed flow cytometric analyses. Cells were stained with specific antibodies for flow cytometry. In brief, cultured hAMCs were washed twice in phosphate-buffered saline (PBS) and harvested with 0.25% trypsin/EDTA (Invitrogen, USA). The cells were then washed with PBS and divided into aliquots for antibody staining. Each aliquot contained approximately 5×10^3^ cells. The antibodies were used to detect the following cell surface antigens: CD44, CD29, CD90, CD73, CD105, CD166, CD45, CD34, and CD14. All antibodies were conjugated with fluorescein isothiocyanate and phycoerythrin. The cells were stained at 4˚C for 30 minutes. After the incubation period, the cells were washed with PBS and resuspended in 500 μL of PBS. Analysis was performed with a FACSCalibur flow cytometer (Becton Dickinson, USA)

## Cytotoxicity investigation

We performed MTT analysis to find the appropriate dose of nanoparticles on the cells. After trypsinization and cell counting, we added 5000 cells added to each well of 96-well microplates. After 24 hours, nanoparticles at concentrations of 0, 25, 50, 75, 100, 125, 150, 175, 200, 400, 600, and 800 μg /mL were added to the wells, and the plates were incubated for 24, 48 and 72 hours. Then, the culture medium that included SPIONs was changed and the medium was washed three times by PBS, each time for 5 minutes in order to remove the nanoparticles from outside the cells and within the wells. Only nanoparticles located inside the cells were allowed to remain. Then, we dissolved 10 mg of tetrazolium powder in 2 ml of PBS. Of this solution, 150 μL was added to each well. The mixture was incubated in a CO_2_ incubator at 37˚C for 4 hours. Next, 100 μL of dimethyl sulfoxide (DMSO, Sigma, USA) was added, and after 10 minutes the light absorption of the resultant solution was observed by an ELISA Reader at 570 to 630 nm. We determined the percentage of viable cells calculated according to the control absorption ratio.

## Intracellular uptake of superparamagnetic iron oxide nanoparticles

We investigated nanoparticles diffusion into the cells by MRI. In order to determine the appropriate concentration for imaging the cells were incubated with various concentrations of nanoparticles (50, 100, 150, 200, 250, 300, 350, 400, 450, 500 μg/ml) and fixed in a 2% agarose gel. The 1.5 T MRI scanners with knee coils were used as follows: echo-times (TE) that ranged from 13 milliseconds to 132, TR of 3000 milliseconds, field of view (FOV) of 23 cm, 8 mm slice thickness, and acquisition matrix of 256×256. 

## Magnetic resonance imaging relaxometry

Relaxometry refers to the measurement of relaxation variables in an MRI in order to determine the specific physical and chemical properties of materials. Solutions of SPION-PEG were prepared in water at iron concentrations of 0.3, 0.63, 1, 1.5, and 2.5 µg/ml for the relaxivity measurements. All measurements were made at room temperature with a 1.5 T MRI clinical scanner (MAGNETOM Avanto, Siemens). T_2_ relaxivity was determined at 1.5 T using spin-echo acquisition that utilized 32 TE, with a range from 13 to 132 milliseconds, and repetition time (TR) of 3000 milliseconds.

The ability of a contrast agent to enhance the proton relaxation rate was determined by the relaxivity (r_i_) that decreased the longitudinal and transverse relaxation times. The equation for the relativity of each contrast agent is:

$$R_{1,2}=\frac{1}{T_{1,2}}={\left(\frac{1}{T_{1,2}}\right)}_{0}\left(R_{0}\right)+r_{1,2}C$$

where T_1_ and T_2_ are longitudinal and transversal relaxations, R_0_ is the relaxation rate without the presence of the contrast agent, C is the concentration (molarity) of the contrast agent and is the relaxivity constant of the agent. The relaxivity of different concentrations of nanoparticles were calculated by linear curve fitting of the relaxation rates $R_{1,2}\left(\frac{1}{T_{1,2}}\right)$ . Therefore, the slope of this curve was r_1, 2_.

T_2_ values were obtained by fitting a logarithmic curve of the mean measured MR signal in a region of interest versus TE. The associated relaxivities (r_2_ in (μg/ml.second)^-1^ were obtained from a linear curve of the slopes of $\frac{1}{T_{1,2}}$ versus the Fe concentration. These calculations were performed using Excel software.

Other scan parameters were as follows: field of view (23 cm), slice thickness (8 mm), and acquisition matrix (256×256). 

## Statistical analysis

The results were presented as mean ± SD from three replicates of each experiment. Statistical analyses were performed with SPSS 21.0 software. Representative data were analyzed for statistical significance by one-way ANOVA with post hoc Bonferroni correction for multiple comparisons. A P value of less than 0.05 was considered statistically significant. In order to obtain relaxometry, the results were analyzed and plots drawn using Radiant application, MATLAB (version 1.0.01), and Microsoft Excel 2010. 

## Ethical considerations

This study received the approval of the Ethics Committee of Iran University of Medical Sciences. 

## Results

### Synthesis and characterization of superparamagnetic iron oxide nanoparticles

**Fig.1:**
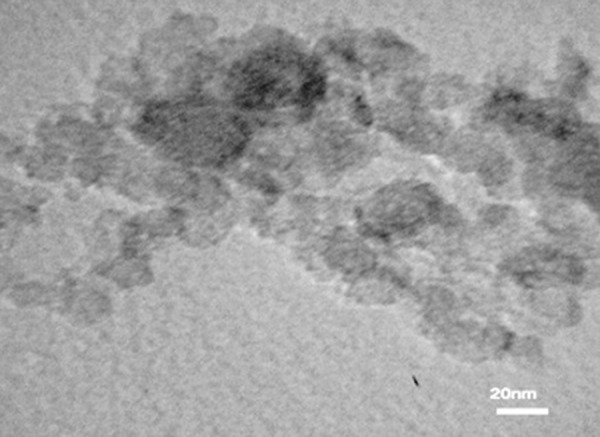
Spherical shape of a synthesized iron oxide nanoparticle under an electron microscope.

**Fig.2:**
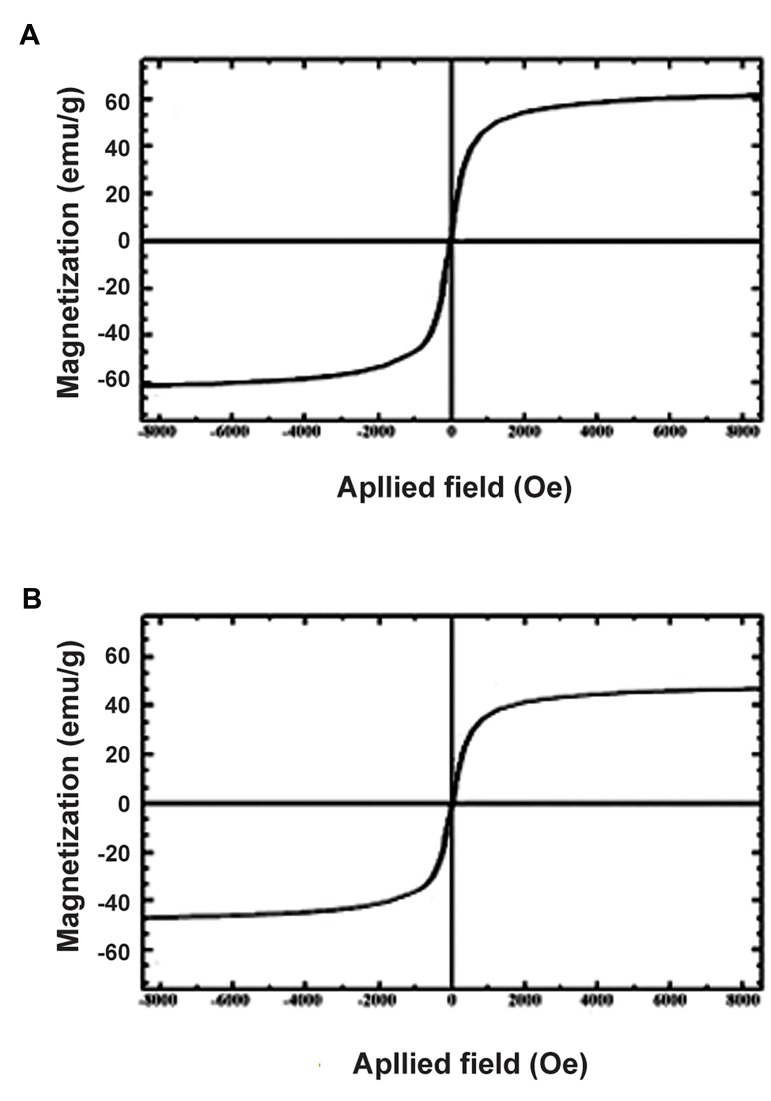
Graph for superparamagnetic behavior of superparamagnetic iron oxide nanoparticles (SPIONs) measured at room temperature. A. Magnetic property of iron oxide nanoparticles without coating and B. Magnetic property of iron oxide nanoparticles coated by polyethylene glycol (PEG).

The resultant SPION samples had a spherical morphology and average particle size of 20 nm (Fig.1). Figure 2 shows the hysteresis loop of nanoparticles as determined by vibrating sample magnetometer VSM that is a scientific instrument that measures magnetic properties. 

These particles showed the superparamagnetism effect. Magnetic properties showed that the coating decreased the magnetic saturation from 60 emu/g to 40 emu/g (Fig.2). 

### Cell culture

**Fig.3:**
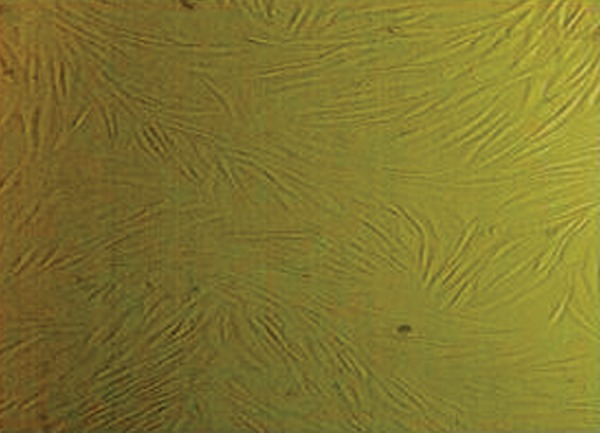
Fifth passage stem cells isolated from the amniotic membrane (×200 magnification).

We successfully isolated and cultured mesenchymal cells from the placental amniotic membrane. At first, the cells had a round shape, which progressed to an elongated shape, and finally a typical fibroblastic morphology (Fig.3). 

### Flow cytometry 

**Fig.4:**
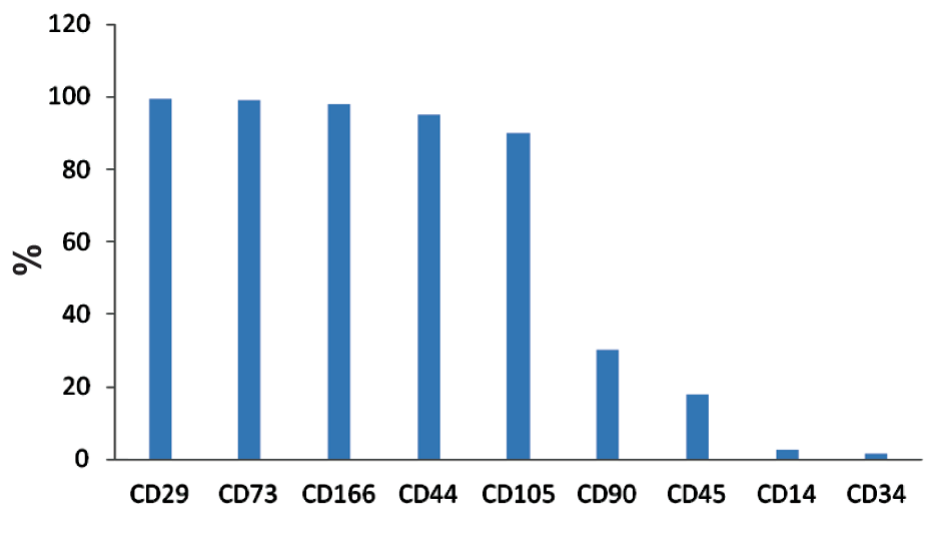
Percentage of markers present in human amniotic membrane mesenchymal stem cells (hAMCs).

After cell extraction, we studied the mean frequency of the CD44, CD29, CD90, CD73, CD105, CD166, CD45, CD34, and CD14 markers (Fig.4). Flow cytometry results showed that the most frequent surface markers of the amniotic membrane stem cells were CD29 (99 ± 1) and CD166 (98 ± 2). The least frequent markers were CD45 (18 ± 11), CD14 (2.5 ± 0.5), and CD34 (1.4 ± 0.7) (Fig.4). 

### Investigation of cytotoxicity

**Fig.5:**
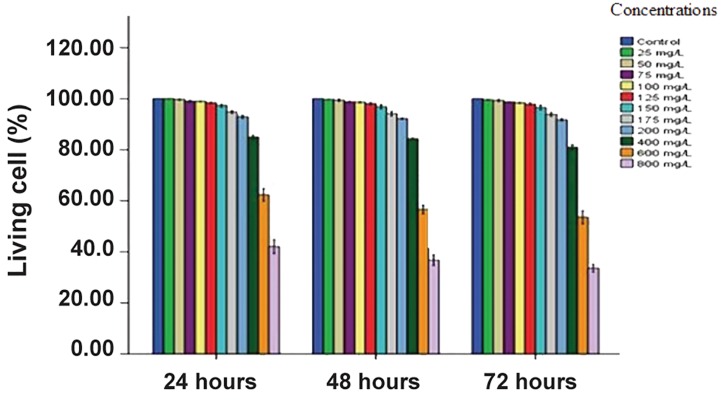
Cell viability of human-derived amniotic membrane stem cells (hAMCs) after exposure to various concentrations of superparamagnetic iron oxide nanoparticles (SPIONs, 0, 25, 50, 75, 100, 125, 150, 175, 200, 400, 600, and 800 μg/mL) for 24, 48 and 72 hours. Data are expressed as means ± SD from three experiments as % of control cells.

MTT results showed 99.96 ± 0.05% cell viability at the lowest concentration (25 μg/mL). Cell viability had a dose-dependent decrease with increased concentrations of the nanoparticles. The nanoparticle cytotoxicities did not significantly differ with control cells during 24 hours at a concentration of 150 μg/mL and 97.26 ± 0.58% viable cells, 48 hours at 125 μg/mL and 98 ± 0.43% viable cells, and 72 hours at 125 μg/mL and 97.86 ± 0.41% viable cells. However the percentage of viable cells after treatment with SPION significantly decreased in hAMCs at 150 μg/ml and higher. A strong association existed with dose and time in terms of SPION toxicity (Fig.5). 

### Magnetic resonance imaging results for the cells

**Fig.6:**
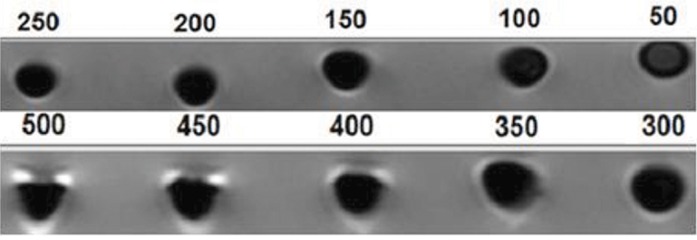
Magnetic resonance imaging (MRI) of stem cells incubated with different concentrations of iron oxide nanoparticles (1.5 T).

In order to examine the nanoparticle effects on cells via MRI, we incubated the cells at different concentrations to show both nanoparticle diffusion and the concentration in which the best images could be obtained. Figure 6 shows that concentrations greater than 300 μg/ml had artifacts that were not good for the imaging process. Therefore, we considered the cells with concentrations below this level. 

### Relaxometry measurements

**Fig.7:**
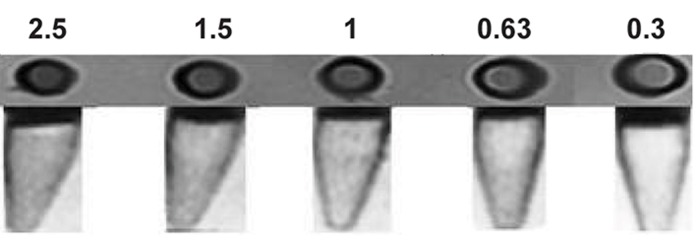
Magnetic resonance imaging (MRI) of different concentrations of iron oxide nanoparticles (1.5 T).

**Fig.8:**
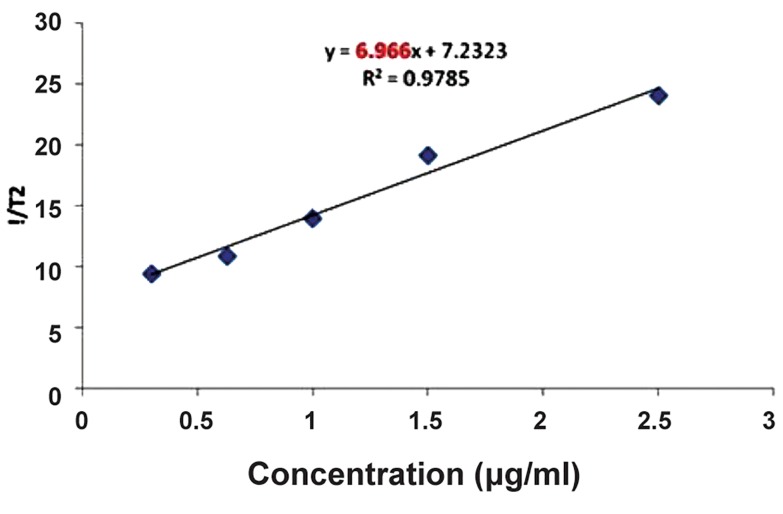
The declining rate of transverse relaxation time in different concentrations of magnetized nanoparticles. According to this formula: $\frac{1}{T_{2}}=\frac{1}{T_{\mathrm{2int}}}+rC$ C; Concentration of iron oxide nanoparticles, 1/T_2_ ; Transverse relaxation time with the presence of nanoparticles, 1/T_2int_ ; Transverse relaxation time of intrinsic water and r; Relaxometry. According to this formula: y=ax+b a; Slope of this curve (6.966) and b; Intercepts of this curve (7.2323).

Figure 7 shows the decrease in transverse relaxation time in terms of concentration. The declining rate of transverse relaxation time will increase with increasing concentrations of nanoparticles, resulting in a signal attenuation of MRI in the presence of the nanoparticles. The relaxometry results have demonstrated that iron oxide nanoparticles coated by PEG showed good relaxometry for MRI and appropriate stability (Fig.8). 

## Discussion

Recently, for cell therapy in addition to the cell type, researchers have expressed a particular interest in their post-injection monitoring. The best approach for monitoring cells is to label them before they are injected into the target tissues (19,20). Currently, substantial interest in using magnetic nanoparticles exists in order to label and track cells because these nanoparticles are efficient, biocompatible and have been approved by the Food and Drug Administration (FDA) (21). In some cases, the cytotoxicity of iron oxide nanoparticles has been reported. This cytotoxicity is associated with the nanoparticle surface coating, morphology, and cell type (12,22,23). In order to obtain a nontoxic nanoparticle, numerous studies have been conducted on various cells treated by these nanoparticles with certain characteristics (24). Berry et al. (25) treated human fibroblast cells with dextran coated iron oxide nanoparticles at a concentration of 50 μg/mL and 15 nm in diameter for three days. They observed decreased cell proliferation and cell death. Pawelczyk et al. (26) treated human macrophage cells with dextran coated iron oxide nanoparticles (100 μg/mL) that were 150 nm in diameter for seven days. In this study, only 20% of the cells survived. Naqvi et al. (27) treated rat macrophage cells with Tween 80 coated iron oxide nanoparticles at concentrations of 25-500 μg/mL and 30 nm in diameter for 1 to 6 hours. 

The results showed that cell damage was related to dose and time. Mahmoudi et al. (28) treated rat cells (L929) with multiple concentrations of polyvinyl alcohol iron oxide nanoparticles (diameter: 82 nm) for three days. They reported a relationship between cell damage to dose and size. Kunzmann et al. (29) treated human macrophage and dendritic cells with multiple concentrations of silica coated iron oxide nanoparticles (diameter: 120-130 nm) for two days. The results showed that cell damage was related to dose and size. Singh Gaharwar and Paulraj (30) treated rat peripheral blood cells with multiple concentrations (7.5-30 mg/kg) of iron oxide nanoparticles that were 30 nm in diameter for one week. They reported that with a decrease in anti-oxidants, the nanoparticles induced oxidative stress and inflammation in the cells. However DNA damage was not significant in terms of cell exposure to the nanoparticles. According to these studies it could be concluded that the size and coating of nanoparticles as well as cell type determined the cytotoxicity levels of the nanoparticles. Many studies showed that iron oxide nanoparticle characteristics such as high relaxivity, high sensitivity and superparamagnetism, which reduce relaxation as well as T_1_ and T_2_ times, made them suitable contrast agents for MRI. Some studies reported that iron oxide nanoparticle cytotoxicity was less than other contrast agents. It was possible to change the magnetic field by manipulating the size and coating of these nanoparticles (19). In the current study, we have sought to examine the effects of iron oxide nanoparticles on hAMC viability because these cells are a substantial source for cell therapy due to their lack of ethical concerns, simple and economical preparation procedure, growth factors, anti-inflammatory factor excretion, and the ability to differentiate into other tissues (1). In order to decrease the cytotoxic effects, we coated these nanoparticles with PEG since various studies have shown that nanoparticles without biocompatible coatings are cytotoxic (12,23). We have treated these cells with multiple concentrations of the nanoparticles. Our results showed that these nanoparticles were not cytotoxic in the range of 150 μg/ml. Above this range the percentage of cell viability considerably diminished compared to the control group. This study used MRI to demonstrate that the nanoparticles were absorbed into the cells through endocytosis without the need for any transfectant agent (30). The relaxometry results showed the efficiency of these nanoparticles as the contrast agent in MRI which has been confirmed by other studies (31,33). In terms of the nanoparticles’ effects on cell survival and MRI results, we found that with appropriate concentrations, iron oxide nanoparticles coated with PEG could be used to label and detect the cells for MRI. 

## Conclusion

In terms of the effects of iron oxide nanoparticles on cell viability, it can be concluded that nanoparticle cytotoxicity increases with increasing the concentration. The results of the current study have shown that PEG-coated iron oxide nanoparticles at suitable concentrations have excellent labeling efficiency and biocompatibility for hAMCs. The data have shown that because of their high R_2_relaxivity, iron oxide nanoparticles can be used as contrast agents in MRI and for cell detection. 
